# Entropy-Based Feature Extraction for Electromagnetic Discharges Classification in High-Voltage Power Generation

**DOI:** 10.3390/e20080549

**Published:** 2018-07-25

**Authors:** Imene Mitiche, Gordon Morison, Alan Nesbitt, Brian G. Stewart, Philip Boreham

**Affiliations:** 1 Department of Engineering, Glasgow Caledonian University, 70 Cowcaddens Rd, Glasgow G4 0BA, UK; 2 Institute of Energy and Environment, University of Strathclyde, 204 George St, Glasgow G1 1XW, UK; 3 Innovation Centre for Online Systems, 7 Townsend Business Park, Bere Regis BH20 7LA, UK

**Keywords:** EMI measurement, partial discharge, entropy, classification, experts system, EMI discharge sources

## Abstract

This work exploits four entropy measures known as Sample, Permutation, Weighted Permutation, and Dispersion Entropy to extract relevant information from Electromagnetic Interference (EMI) discharge signals that are useful in fault diagnosis of High-Voltage (HV) equipment. Multi-class classification algorithms are used to classify or distinguish between various discharge sources such as Partial Discharges (PD), Exciter, Arcing, micro Sparking and Random Noise. The signals were measured and recorded on different sites followed by EMI expert’s data analysis in order to identify and label the discharge source type contained within the signal. The classification was performed both within each site and across all sites. The system performs well for both cases with extremely high classification accuracy within site. This work demonstrates the ability to extract relevant entropy-based features from EMI discharge sources from time-resolved signals requiring minimal computation making the system ideal for a potential application to online condition monitoring based on EMI.

## 1. Introduction

The High-Voltage (HV) power supply industry involves the use of generators, motors, transformers, transmission lines and cables that jointly contribute to generate electrical power. The effective operation of these assets is important as any failure in one part of the system may have significant and often drastic consequences in terms of loss of production, induced costs, safety and down-time. Early detection or prediction of fault occurrence along with fault source identification would potentially avoid such dramatic consequences as appropriate risk mitigation measures could be put in place. Electrical power assets such as generators, motors and transformers are susceptible to electrical insulation problems [1] which can be manifested in the form of faults such as Partial Discharge (PD) and Arcing. PD is a sign of insulation degradation which is considered harmful for an asset, as once present it becomes a further source of accelerated insulation degradation [2]. As such, PD monitoring is seen as a significant tool for electrical insulation condition assessment [3]. PD can be assigned several different PD categories such as Surface Discharge, and Internal Discharge [4]. An arcing fault is also an electrical discharge through an insulating medium [5]. An arc occurs when electric current is transmitted through a gaseous or liquid environment. These latter two faults commonly arise in power transformers [6]. Early detection of these faults helps to take decisions on an asset’s maintenance which reduces the high cost of purchasing new replacement equipment as well as avoiding potential statutory fines and civil complaints [4]. Identifying PD faults may be achieved from appropriate PD captured data using pattern recognition and classification techniques along with appropriate feature extraction methods [7]. This approach for PD classification has been investigated in the literature by many researchers within power systems [8,9]. In [10] the authors extracted image-oriented features from phase resolved plots of PD signals and employed different classifiers for pattern recognition, e.g., Fuzzy k-Nearest Neighbour Classifier (FkNNC), Back-Propagation Neural Network (BPNN) and Support Vector Machine (SVM). Classification of multiple PD sources (Void, Air-Corona and Oil-Corona) was achieved in [11] using statistical quantities, such as mean deviation, quartile deviation etc., calculated on the Phase Resolved PD (PRPD) patterns and classified with a Probabilistic Neural Network (PNN). A comparison between PNN and SVM classification of PD sources in [12] revealed that SVM slightly outperforms PNN in terms of classification accuracy. From the research, PD analysis and classification revealed that different discharge sources have a unique fingerprint that can be quantified with the help of feature extraction techniques.

For many years, the Electro-Magnetic Interference (EMI) method has been successfully applied to diagnose generator and transformer faults (e.g., [13,14]). This method differs from other fault diagnosis methods in that EMI looks at the frequency and time-domain measurements of captured signals using The Comite International Special des Perturbations Radioelectriques (CISPR) 16 Standard approach. In CISPR 16, different bandpass filters are used to measure the signal energy over different frequency ranges. The presence of frequency bands in a CISPR 16 spectrum is indicative of different types of generator faults, with the most important fault frequency bands existing well below 100 MHz (see [13,14]). Once a frequency band and an associated frequency of interest has been selected in an EMI spectrum, the audio time-domain signal at the selected EMI frequency is captured and reviewed by an EMI expert for “expert” fault classification, e.g., Arcing, Corona, Exciter, Data Modulation, PD etc. [15]. Currently there is a limited amount of work investigating the use of intelligent pattern recognition or signal fault classification methods for time-domain EMI sources within power system equipment. The aim of this work is to assess the potential for the use of Entropy-based methods in EMI-based condition monitoring. To this end, this paper applies four simple and computationally low feature extraction methods based on entropy along with Multi-Class SVM (MCSVM) and Random Forests (RF) classifiers with the aim of separating and classifying multiple EMI time-domain signals. The measurements investigated in this paper were collected from various HV power stations which were identified to contain EMI faults based on EMI analysis from field experts. More details regarding the data and classification are discussed later in this paper.

The paper is organised as follows. Section 2 discusses the EMI method of monitoring and capturing PD signals in more detail. Section 3 introduces the feature extraction and classification techniques employed. Section 4 describes the data measurement process and classification experimental set-up. Section 5 presents the results from measured signals; evaluation of the performance of the classification approach in relation to the segmented size of time domain data collected is also considered. The last section draws conclusions on the work and provides suggestions on future work.

## 2. EMI Monitoring

An EMI measurement of power equipment is carried out as follows. Using a High-Frequency-Current-Transformer (HFCT), signals are measured on the neutral earth cable of, for example a generator or transformer. The signals are usually recorded with a PD Surveyor 200 (PDS200) instrument that functions under the CISPR16 standard [16] for EMI filter types. CISPR 16 specifies the use of a quasi-peak detector implemented with different filter bandwidths over the range of frequencies to be measured. For example, the B filter operates over 150 kHz–30 MHz, with a 6 dB bandwidth of 9 kHz, the C filter from 30–300 MHz with a 6dB bandwidth of 120 kHz. The important feature of PDS200 is it acts as a radio receiver with the ability to capture and analyse Radio Frequency Interference (RFI) as well as EMI radiations in the lower frequency range [50 kHz–1 GHz]. This provides a frequency spectrum of unique signature for each fault or condition [13,14,17]. The PDS200 looks at signals based on the CISPR16 bandwidths (e.g., 9 kHz, 120 kHz) and moves through the frequency spectrum making appropriate power filtered response measurements at each frequency. Quasi-peak measurements are made over a 1 s time-period at each selected frequency. For time domain signals the instrument permits any frequency between 150 kHz to 100 MHz to be selected e.g., where the maximum envelope energy exists. It then makes the slower demodulated response measurement which is sampled at 24 kHz. EMI time-domain signals can also be retrieved at the frequencies of interest for audio examination by EMI experts in order to determine or classify the nature of the fault [15]. This ability is based on a wealth of previous experiences of audio fault assessment and forensic confirmation. The limitation of this method is that it relies completely on the availability of an expert to ascertain the fault diagnosis. However, an experienced expert with a modest training is capable of identifying many of the common faults related to EMI signals and the experts involved in this work have extensive accumulated experience of fault diagnosis and forensic confirmation. This work attempts to capture this expert knowledge and to use this as a foundation for initial training of fault recognition algorithms and automated classification for EMI time-domain signals. To achieve this, a method of feature extraction and classification requires to be implemented, which is presented in the following section.

## 3. Description of Employed Algorithms

**Figure 1:**
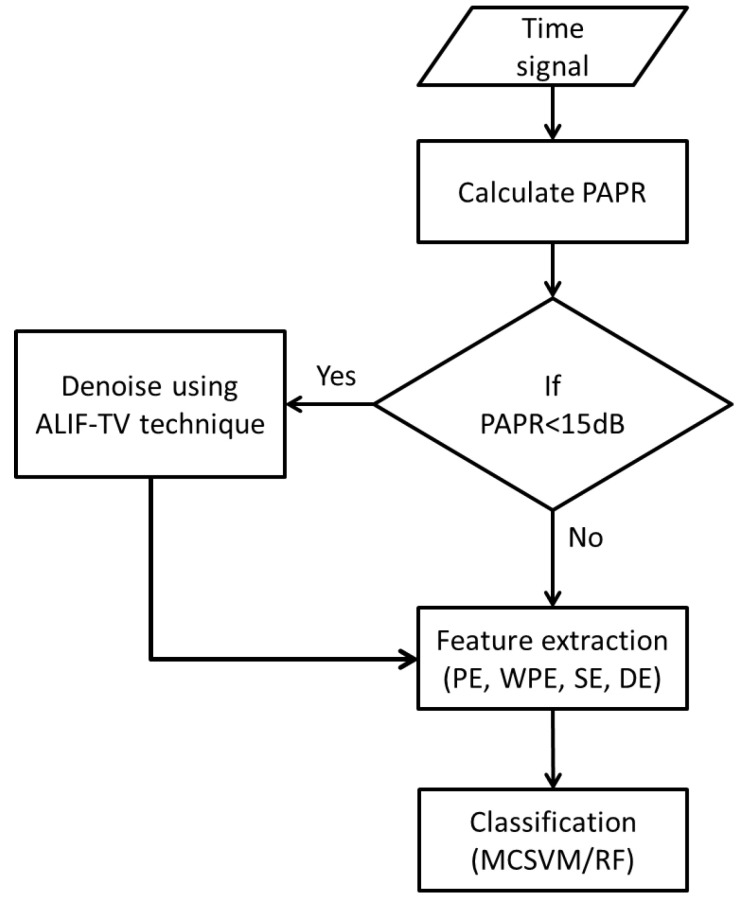
Flow diagram of the overall proposed algorithm.

The experiment design is summarised in the flow diagram in Figure 1. In this work, denoising approach is implemented, on the EMI time signals, as preprocessing. It is important to apply this step to noisy signals only as denoising of a noise free signal may result in distortion which will subsequently destroy the important signal information. Thus, Peak to Average Power Ratio (PAPR) is used as a decision metric to whether the signal should be denoised.

The PAPR, defined in Equation (1), is calculated for each time signal and compared against a threshold of 15 dB. The choice of this threshold is based on evaluation of the whole experiment using different thresholds in the range of [10–20 dB] with a step of one. The main reasons of selecting this range of PAPR are under the assumption of the following. The discharge impulses such as PD have a much higher peak amplitude than noise amplitude. Therefore, a high PAPR indicates that the signal contains little noise. The common noise found in EMI measurement is similar to the one observed in telecommunication system, which typically has a PAPR that varies between [10–20 dB] [18].

(1) $$\mathrm{PAPR}=10\log_{10}\left(\frac{|x_{peak}{|}^{2}}{x_{rms}^{2}}\right)$$

Relevant information is then extracted from the denoised or noise-free signals by means of Permutation Entropy (PE), Weighted Permutation Entropy (WPE), Sample Entropy (SE) and Dispersion Entropy (DE). The four entropies are embedded into a feature vector per signal instance, which is implemented in MCSVM and RF classifiers. The employed denoising, feature extraction and classification algorithms are explained in detail as follows.

### 3.1. Signal Denoising

The employed algorithm is called Adaptive Local Iterative Filtering combined with Total Variation (ALIF-TV) and was proposed in [19] to effectively mitigate noise in field measured EMI signals. The main idea of this method is to first decompose the signal, using ALIF, into its different frequency components also called Intrinsic Mode Function (IMF), then employ TV thresholding to reduce noise towards zero. The denoised signal is reconstructed as the sum of the components after thresholding. First, we denote scalars by lower case, vectors by bold lower case and matrices by bold upper case. The mathematical framework of this algorithm is described as follows.

Let the time series signal, y(n);n=1,...,N, be decomposed into *K* number of IMFs plus a residual trend r(n) as:

(2) $$y\left(n\right)={\mathrm{imf}}_{1}\left(n\right)+{\mathrm{imf}}_{2}\left(n\right)+...+{\mathrm{imf}}_{K}\left(n\right)+r\left(n\right).$$

The IMFs are obtained through filtering as:

(3) $$y_{j+1}\left(n\right)=y_{j}\left(n\right)-\sum_{-l_{j}\left(n\right)}^{l_{j}\left(n\right)}h_{j}\left(n,v\right)\cdot y_{j}\left(n+v\right)$$

where $h_{j}\left(n,v\right)\in R$, $n\in [-l_{j}\left(n\right),l_{j}\left(n\right)]$ are low pass filter coefficients at point *n* with length of $2l_{j}\left(n\right)+1$. Equation (3) is iterated until it converges to obtain the IMFs, this is known as inner iteration. Outer iteration is performed and converges when the residual trend is obtained. This is delineated when the number of points in $y_{j}\left(n\right)$ is continuously reduced while the number of iterations increases. The ALIF algorithm is embedded with TV to obtain the IMFs of a noisy signal y(n) by the function

(4) $$A\left(y\left(n\right)\right)=\left[\begin{array}{c} {\mathrm{imf}}_{1}\left(n\right)\\ {\mathrm{imf}}_{2}\left(n\right)\\ \vdots \\ {\mathrm{imf}}_{K}\left(n\right) \end{array}\right]=W.$$

Each IMF in W is denoised using TV thresholding. Here the thresholding is not applied to the residual. The denoised signal is reconstructed by summation of the denoised IMFs plus the residual. The TV of a signal y(n) is defined as follows:

(5) $$\mathrm{TV}\left(y\left(n\right)\right):={\left||\mathrm{Dy}\left(n\right)|\right|}_{1}$$

where $\left|\right|\cdot {\left|\right|}_{1}$ is the $l_{1}$-norm and D is the first order difference matrix defined as:

(6) $$D=\left[\begin{array}{ccccc} -1 & 1 & & & \\ & -1 & 1 & & \\ & & \ddots & \ddots & \\ & & & -1 & 1 \end{array}\right].$$

The ALIF-TV minimises the noise in the IMFs ($\hat{W}$) by solving the following non-convex optimisation problem:

(7) $$\hat{W}=arg\underset{W}{min}\{F\left(W\right)=\frac{1}{2}||A\left(y\left(n\right)\right)-{W||}_{2}^{2}+\sum_{j}\lambda_{j}\phi \left(W_{j};\alpha_{j}\right)+\beta ||DB\left(W\right){\left|\right|}_{1}\}$$

where $\left|\right|\cdot {\left|\right|}_{2}^{2}$ is the $l_{2}$-norm, B(W) is the reconstruction function defined as follows:

(8) $$B\left(W\right)=\left(\sum_{j=1}^{K}W_{j}\right)+r\left(n\right)$$

ϕ is a penalty function [20] with parameter $\alpha_{j}=\frac{1}{\lambda_{j}}$, and the regularisation parameters β and λ are defined as follows.

(9) $$\beta =\left(1-\eta \right)\sqrt{N}\frac{\sigma }{4}$$

(10) $$\lambda =\left(2.5\eta \sigma \right)/\sqrt{2^{j}}$$

Here, η=0.95 is selected according to [20] to balance the weight between the TV and IMFs in the optimisation problem. The variance of the noise σ is estimated, using the popular Donoho Median Absolute Deviation (MAD) [21], in the following expression:

(11) $$\sigma =\frac{MAD\left(y(n)\right)}{0.6745}.$$

The non-convex optimisation problem is solved by means of the split augmented Lagrangian shrinkage algorithm [22]. Variable splitting is a straightforward approach that involves the creation of a new variable, here U, which serves as the argument of $f_{2}$ under the constraint that g(W)=U. This results in a constrained problem of Equation (7) as:

(12) $$\hat{W}=arg\underset{W}{min}\left\{f_{1}\left(W\right)+f_{2}\left(U\right)\right\}$$

where U=W initially, and

(13) $$f_{1}\left(W\right)=\frac{1}{2}||A\left(y\left(n\right)\right)-{W||}_{2}^{2}+\sum_{j}\lambda_{j}\phi \left(W_{j};\alpha_{j}\right)$$

(14) $$f_{2}\left(U\right)={\beta ||DB\left(W\right)||}_{1}.$$

The augmented Lagrangian is then implemented as follows:

(15) $$L\left(W,U,\mu \right)=f_{1}\left(W\right)+f_{2}\left(U\right)+\frac{\mu }{2}||U-W-{V||}_{2}^{2}$$

where μ is a step-size parameter set to 1 according to [20], and V=0 in the initial iteration. Hence, Equation (12) is solved in three main steps of sub-problems (Equations (16) to (18)) iteratively:

(16) $$W=arg\underset{W}{min}\{f_{1}\left(W\right)+\frac{\mu }{2}||U-W-{V||}_{2}^{2}\}$$

(17) $$V=arg\underset{W}{min}\{f_{2}\left(V\right)+\frac{\mu }{2}||U-W-{V||}_{2}^{2}\}$$

(18) V=V-(U-W).

Please note that the functions $f_{1}$ and $f_{2}$, and the sub-problems in Equations (16) and (17) are strictly convex. When convergence of the iterative algorithm is achieved, using the theory in [23], this solves Equation (7) and the denoised signal reconstruction is obtained by:

(19) $$\hat{y}\left(n\right)=B\left(\hat{W}\right).$$

### 3.2. Entropy Measures

#### 3.2.1. Permutation Entropy (PE)

PE was introduced by Bandt and Pompe [24] to assess the complexity in time series data based on the comparison of successive adjacent values which are mapped to ordinal patterns. The advantage of ordinal patterns helps in providing more resilience to low frequency artefacts. This makes it suitable for measuring real-world, noisy and chaotic time series signals [25]. PE is derived from Shannon’s entropy which is a measure of the level of information contained in a data set [26]. Bandt and Pompe combined the entropy concept with symbolic dynamics to create a simple, fast to compute, robust and stable measure of regularity in short time series and to overcome classical entropy method limitations, including the requirement of long data sets and high computational cost [27]. The PE algorithm is described in [28] as follows. Based on a given time series {x(1),x(2),...,x(N)}, vectors x(j);j=1,2,...,N-(m-1) are constructed as:

(20) x(j)={x(j),x(j+1),...,x(j+(m-1))}.

The sequence in Equation (20) is arranged to provide components in increasing order as follows:

(21) $$\;\{x\left(j+\left(i_{1}-1\right)\right)\leq x\left(j+\left(i_{2}-1\right)\right)\leq ...\leq x\left(j+\left(i_{m}-1\right)\right)\}.$$

If two successive components are equal i.e., $x\left(j+\left(i_{1}\right)\right)=x\left(j+\left(i_{2}\right)\right)$ for $i_{1}\leq i_{2}$, then their positions can be rearranged to $x\left(j+\left(i_{1}\right)\right)\leq x\left(j+\left(i_{2}\right)\right)$. Next a different symbol series s(l) is calculated for each time series x(i) as:

(22) $$\;s\left(l\right)=\left(i_{1},i_{2},...,i_{m}\right)$$

where l=1,2,...,k(k=m!). That is, there will be m! different symbol series or permutations $\pi_{n}$. The probability $p(\pi_{n}^{m})$ of each symbol sequence s(l) is then calculated mathematically as:

(23) $$\;p\left(\pi_{n}^{m}\right)=\frac{\sum_{j\leq N}^{1}u:type\left(u\right)=\pi_{n}\left(x_{n}^{m}\right)}{\sum_{j\leq N}^{1}u:type\left(u\right)\in \Pi \left(x_{n}^{m}\right)}.$$

The mapping of the symbol s(l) to the ordinal pattern is denoted as type(·) and the m! symbols ${\left\{\pi_{n}^{m}\right\}}_{n}^{m!}$ are denoted as Π. The indicator function $1_{a}\left(u\right)$ of a set a is defined as:

$$\;1_{a}\left(u\right)=\left\{\begin{array}{c} 1\;if\;u\in a\\ 0\;if\;u\notin a \end{array}\right.$$

Finally the value of PE can be estimated using the formula:

(24) $$\;\mathrm{PE}=-\sum_{n=1}^{m!}p\left(\pi_{n}^{m}\right)\times \log\left(p\left(\pi_{n}^{m}\right)\right).$$

PE should be suitable to quantify the characteristics contained in EMI signals since they are by nature non-stationary and complex to analyse.

#### 3.2.2. Weighted Permutation Entropy (WPE)

WPE is derived from PE except that in WPE patterns with same amplitude variations are grouped within the same pattern [29]. Given the time series data {x(1),x(2),...,x(N)} with length *N*, the time data is mapped to a space of *m* dimension and a delay τ in order to obtain the vector $x{\left(j\right)}^{m,\tau }=\left\{x\left(j\right),x\left(j+\tau \right),...,x\left(j+\left(m-1\right)\tau \right)\right\};j=1,2,...,N-\left(m-1\right)\tau$. m! Permutation patterns $\pi_{i}$ are created with an embedded dimension of *m*. Each vector x(j) is compared to a permutation pattern $\pi_{i}$ in the *m* dimensional space and is weighted by a weight $w_{j}$ which is obtained from the variance of each neighbour’s vector $x{\left(j\right)}^{m,\tau }$ as:

(25) $$w_{j}=\frac{1}{m}\sum_{k=1}^{m}{\left(x\left(j+\left(k-1\right)\tau \right)-\bar{x{\left(j\right)}^{m,\tau }}\right)}^{2}$$

where $\bar{x{\left(j\right)}^{m,\tau }}$ is the mean of x(j) which is defined as:

(26) $$\;\bar{x{\left(j\right)}^{m,\tau }}=\frac{1}{m}\sum_{k=1}^{m}x\left(j+\left(k-1\right)\tau \right).$$

The weighted probabilities of occurrence for each pattern group $\pi_{i}^{m,\tau }$ are estimated as:

(27) $$\;p_{w}\left(\pi_{i}^{m,\tau }\right)=\frac{\sum_{j\leq N}1_{u:type\left(u\right)=\pi_{i}}\left(x{\left(j\right)}^{m,\tau }\right)\times w_{j}}{\sum_{j\leq N}1_{u:type(u)\in \Pi }\left(x{\left(j\right)}^{m,\tau }\right)\times w_{j}}.$$

WPE can then be estimated based on Shannon Entropy [26] as:

(28) $$\mathrm{WPE}=-\sum_{i:\pi_{i}^{m,\tau }\in \Pi }p_{w}\left(\pi_{i}^{m,\tau }\right)\times \log\left(p_{w}\left(\pi_{i}^{m,\tau }\right)\right)$$

It was demonstrated in [30] that WPE is effective in the analysis of non-linear time series, which is a motivation to exploit it in this paper as a feature extraction technique for discharge sources since they are by nature non-stationary. Bandt and Pompe [24] suggest embedded dimension *m* between 3 and 7. A high value of *m* provides more patterns which increases memory and computation. Since an aim in this work is to employ low computationally complex methods that could potentially be implemented in an instrument, the minimum m=3 is chosen for both PE and WPE.

#### 3.2.3. Sample Entropy (SE)

SE is an entropy-based complexity measure for time series data [31] which directly measures the degree of randomness and inversely measures the degree of orderliness. The overall idea in SE is to determine the conditional probability that *m* consecutive data points, which are similar within a range or tolerance *r*, will preserve this similarity if the next data point is added, given that self-matches are ignored [32]. The steps for SE calculation are described as follows.

For the input time series {x(1),x(2),...,x(N)} of length *N*, vectors $(z_{m}\left(j\right));j=1,...,N-m$ are constructed such that:

(29) $$\;z_{m}\left(j\right)=x(j+k);\;0\leq k\leq m-1.$$

The maximum difference between the magnitude of two vectors determines the distance between them, and this distance is compared against a tolerance *r* providing a proportion of two vectors, $x_{m}\left(j\right)$ and $x_{m}\left(i\right),i\neq j$ with length *m*, is defined as

(30) $$\;s_{j}^{m}=\frac{1}{N-m-1}\sum_{i=1,i\neq j}^{N-m}\Theta (r-||z_{m}\left(j\right)-x_{m}\left(i\right)||).$$

On the other hand, the proportion of two vectors, $x_{m}\left(j\right)$ and $x_{m}\left(i\right),i\neq j$ with length m+1, is defined as:

(31) $$\;s_{j}^{m+1}=\frac{1}{N-m-1}\sum_{i=1,i\neq j}^{N-m}\Theta (r-||z_{m+1}\left(j\right)-x_{m+1}\left(i\right)||)$$

where

(32) $$\Theta \left(x\right)=\left\{\begin{array}{c} 0,\;x<0\\ 1,\;x\geq 0 \end{array}\right.$$

Next, the grouping average is calculated as:

(33) $$\;u^{m}=\frac{1}{N-m}\sum_{j=1}^{N-m}s_{j}^{m}$$

(34) $$\;u^{m+1}=\frac{1}{N-m}\sum_{j=1}^{N-m}s_{j}^{m+1}.$$

Finally, SE values are calculated using the expression:

(35) $$\mathrm{SE}=-\log\left(\frac{u^{m+1}}{u^{m}}\right).$$

The recommended embedded dimension *m* and tolerance level *r* values are *m* = 2, r=0.2×σ where σ is the standard deviation of the time series [32]. Thus, these parameters are used in this work.

#### 3.2.4. Dispersion Entropy (DE)

DE was introduced in [33] to overcome PE and Sample Entropy (SE) limitations. SE is slow in computation especially for long time series, whereas PE disregards information of the amplitude values mean and amplitude variations [34]. DE measure can be obtained as follows. Given the time series signal {x(1),x(2),...,x(N)}, with length of *N*, let x be mapped to y={y(1),y(2),...,y(N)} using the Normal Cumulative Distribution Function (NCDF) which is defined as:

(36) $$y=F\left(x|\mu ,\sigma \right)=\frac{1}{\sigma \sqrt{2\pi }}\int_{-\infty }^{x}e^{\frac{-{\left(t-\mu \right)}^{2}}{2\pi \sigma^{2}}}dt$$

NCDF values are the probabilities that a random variable from the normal distribution, with mean (μ) and standard deviation (σ) of the total signal x, is less than $x_{j}$ value from the time series x. Next, each y(j);j=1,...,N is assigned a class from 1 to *c* linearly as follows.

(37) $$\;z_{j}^{c}=\mathrm{round}\left(c.y\left(j\right)+0.5\right)\;j=1,...,N.$$

This provides *N* members of the classified time series. Here other linear or non-linear methods can also be employed. Embedding vectors $z_{i}^{m,c}$ with dimension *m* and time delay τ are then created:

(38) $$z_{i}^{m,c}=\left\{z_{i}^{c},z_{i+d}^{c},...,z_{i+(m-1)d}^{c}\right\};\;i=1,2,...,N-\left(m-1\right)\tau .$$

The latter is mapped to a dispersion pattern $\pi_{v_{o}v_{1}...v_{m-1}}$, among $c^{m}$ possible dispersion patterns, in that $v_{0}=z_{i}^{c},v_{1}=z_{i+d}^{c},...,v_{m-1}=z_{i+(m-1)d}^{c}$.

The dispersion probability of occurrence for each pattern is then calculated as follows.

(39) $$p\left(\pi_{v_{o}v_{1}...v_{m-1}}\right)=\frac{\sum_{i\leq N-(m-1)\tau }1_{u:type\left(u\right)=\pi_{v_{o}v_{1}...v_{m-1}}}\left(z_{i}^{m,c}\right)}{N-(m-1)\tau }.$$

Finally, the DE value is obtained based on the Shannon entropy formula as follows.

(40) $$\;\mathrm{DE}=-\sum_{1}^{c^{m}}p\left(\pi_{v_{o}v_{1}...v_{m-1}}\right).\log\left(p\left(\pi_{v_{o}v_{1}...v_{m-1}}\right)\right).$$

The calculated DE value provides the level of spreading in a time series, which may be informative on the features of the different discharge signals. In this paper, the standard values m=2 and c=3 are followed [33].

### 3.3. Classification Algorithms

Supervised classification process could be outlined as follows. Provided a labelled training data set, the classifier model learns a mapping space which is able to predict the true labels of an unseen testing data set. In this paper, two different classification algorithms, MCSVM and RF, are employed and discussed as follows.

#### 3.3.1. Support Vector Machine (SVM) and Multi-Class SVM (MCSVM)

**Figure 2:**
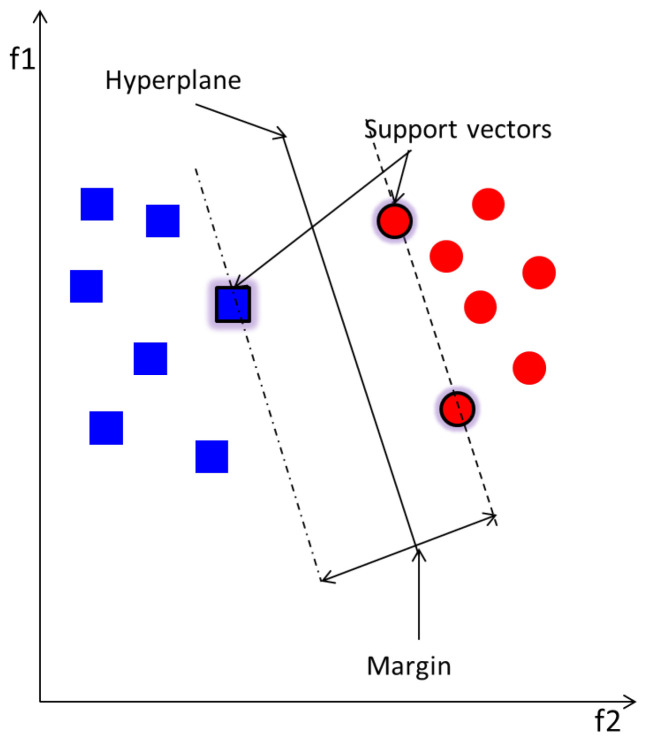
Support Vector Machine (SVM) linear separation.

SVM is used in this work as a binary classifier that groups separately data from two classes only in the feature space by means of a hyperplane, whose distance from the nearest point of each class determines the margin [35]. Ideal SVM performance requires wide margin and thus an optimum separation between the classes. The basic SVM separates two classes using a linear kernel function as illustrated in in Figure 2. There exist other kernels, such as quadratic, polynomial and Radial Basis Function (RBF), to adapt the nature of data. Optimisation techniques are recommended to choose the best kernel function, along with its parameter, to employ for a high performance. Here, a grid search method [36] is employed as an optimisation technique. This involves multiple training and testing of the MCSVM with a grid of all possible kernel functions and their parameters.

The implementation theory of SVM can be summarised as follow: Let x be the data input and y the associated labels with length *L*. It is assumed that the data points belong to two classes “1”and “-1”. Each data point is non-linearly mapped to a feature space separated by a hyperplane with the basic geometric equation:

(41) f(x)=w·x+b=0

where *b* is a scalar and w is L-dimensional vector parameters that play an important role to determine the hyperplane position. If b=0, the hyperplane will pass by the origin. Otherwise, the margin is created or increased. The parallel hyperplanes that separate the two different data classes are defined as w·x+b=1 and w·x+b=-1 for the first and second class respectively. The hyperplane is obtained as a solution to the optimisation problem in Equation (42), while considering the noise slack variable $\zeta_{i}$ which determines the range to which the samples overstep the margin, and the error penalty *c* which represents the trade-off between maximisation of the margin and classification error on training phase.

(42) $$\;min\frac{1}{2}{\left||w|\right|}^{2}+c\sum_{i=1}^{M}\zeta_{i}$$

$$\mathrm{Subject}\;\;\mathrm{to}\left\{\begin{array}{cc} y_{i}\left(w^{T}.x_{i}+b\right)\geq 1-\zeta_{i}\\ \zeta_{i}\geq 0, & i=1,...,M \end{array}\right.$$

where $\zeta_{i}$ denotes the distance between the margin and the data point which is in error. The calculation of Equation (42) is simplified and solved by converting it to a Lagrangian problem which is explained in more detail in [37]. This introduces an $\alpha_{i}$ parameter which expresses w in solving Equation (42). The solution yields to a non-linear decision function expressed as:

(43) $$\;f\left(x\right)=\mathrm{sign}(\sum_{i,j=1}^{M}\alpha_{i}y_{i}\left(x_{i}x_{j}\right)+b).$$

The issues that may arise from a learning process in SVM with high dimension feature space are data over-fitting and computational errors. Over-fitting can be solved by introducing a kernel function Φ(x) which performs a dot product of the feature space. The definition of this kernel function can be found in [35] . This is the case of non-linear mapping using a kernel function as discussed earlier for Figure 2. The non-linear vector function $\Phi \left(x\right)=(\phi_{1}\left(x\right),...,\phi_{l}\left(x\right))$, with feature space of dimension *l*, is implemented and the decision function in Equation (43) can be reformulated as:

(44) $$\;f\left(x\right)=\mathrm{sign}(\sum_{i,j=1}^{M}\alpha_{i}y_{i}\left(\Phi^{T}\left(x_{i}\right).\Phi \left(x_{j}\right)\right)+b).$$

To classify more than two classes, the MCSVM approach is exploited by employing the one-against-one (OAO) strategy. This involves training each of k(k-1)/2 models on two classes, *p* and *q*, as a normal binary classification provided that *k* is the total number of classes. A “Max-Win” voting method is employed for prediction. The vote for a predicted class is increased by one when the unseen data sample is close to that class, and the one that receives the highest vote is considered to be the predicted class of this data sample.

#### 3.3.2. Random Forests (RF)

The RF classifier is a group of decision trees that are trained on random features sets of labelled data. The randomness property provides de-correlated trees. The model is formed by an ensemble of components including weak learners and leaf predictor type. The motivation for using RF revolves around its properties. The main one is the ability to handle more than two classes. Furthermore, the RF technique is efficient because of its low model variances (over-fitting) and its parallelism structure. These advantages are a motivation to employ RF as an additional classifier, then compare its performance against MCSVM.

In brief, training of an RF classifier is achieved in the following steps.

1. At an initial node, randomly choose P feature instances from the overall instances Q presented to the classifier, where P is much smaller than Q.
2. Calculate the best split point using Information Gain defined as:
  (45) $$I=H\left(s\right)-\sum_{i\in \{1,2\}}|\frac{s^{i}}{s}|H\left(s^{i}\right)$$
  where H(s) is the Shannon Entropy [26] of the node s and $s^{i}$ is the child node.
3. Using the best split point, divide the main node into daughter nodes and reduce the number of feature instances along the nodes.
4. Repeat steps 1 to 3 until a maximum depth l=5 is reached.
5. Repeat steps 1 to 4 for K=500 trees of the model. The more trees that are employed then the higher the achieved performance.

**Figure 3:**
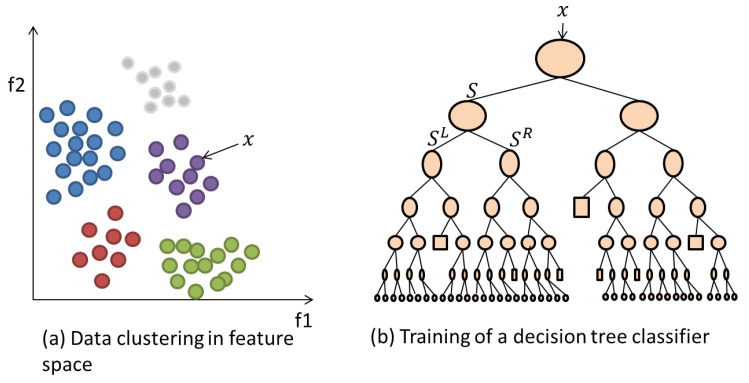
(**a**) Example feature space representation of data instances that belong to 5 different classes (colours). (**b**) An example decision tree classifier architecture.

Let a training instance, from a training set $Q={\left\{\left(x_{i},y_{i}\right)\right\}}_{i=1}^{n}$, be a vector of *d* dimension features x=(x(1),...,x(d) with its associated labels y. The overall RF model is an ensemble of *K* weak classifiers $h=\{h_{1}\left(x\right),...,h_{K}\left(x\right)\}$ where each $h_{k}$ is a decision tree defined as:

(46) $$h_{k}\left(x\right)=h\left(x|\gamma_{k}\right)$$

provided that $\gamma_{k}=\gamma_{k1},...,\gamma_{kp}$ is a set of parameters that decide the variables at which the nodes are split, tree structure etc. These parameters are optimised during the training phase by maximising the Information Gain objective function $\gamma_{j}$ as:

(47) $$\gamma_{j}^{*}=arg\underset{\gamma_{j}}{max}\left\{I_{j}\right\}$$

where $I_{j}$ is the Information Gain at node *j*. The mathematics of this algorithm are detailed further in [38]. Figure 3 illustrates the training of a single tree in the RF on a training data set as discussed in the previous steps. The data/label pair instances are utilised to optimise the parameters within each node in order to provide a trained model. The testing phase involves label prediction of the unseen data features based on the rules and parameters generated during the RF model creation. Each trained decision tree $h_{k}$ within the model provides a prediction output and the highest voted label among all trees is considered to be the final predicted label.

## 4. Experimental Set-Up

### 4.1. EMI Signals Measurement

**Figure 4:**
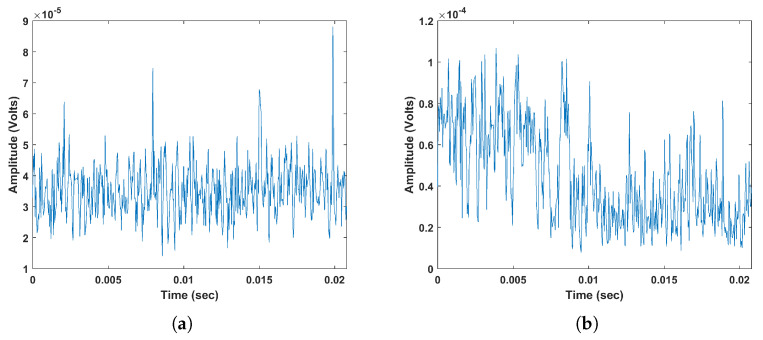

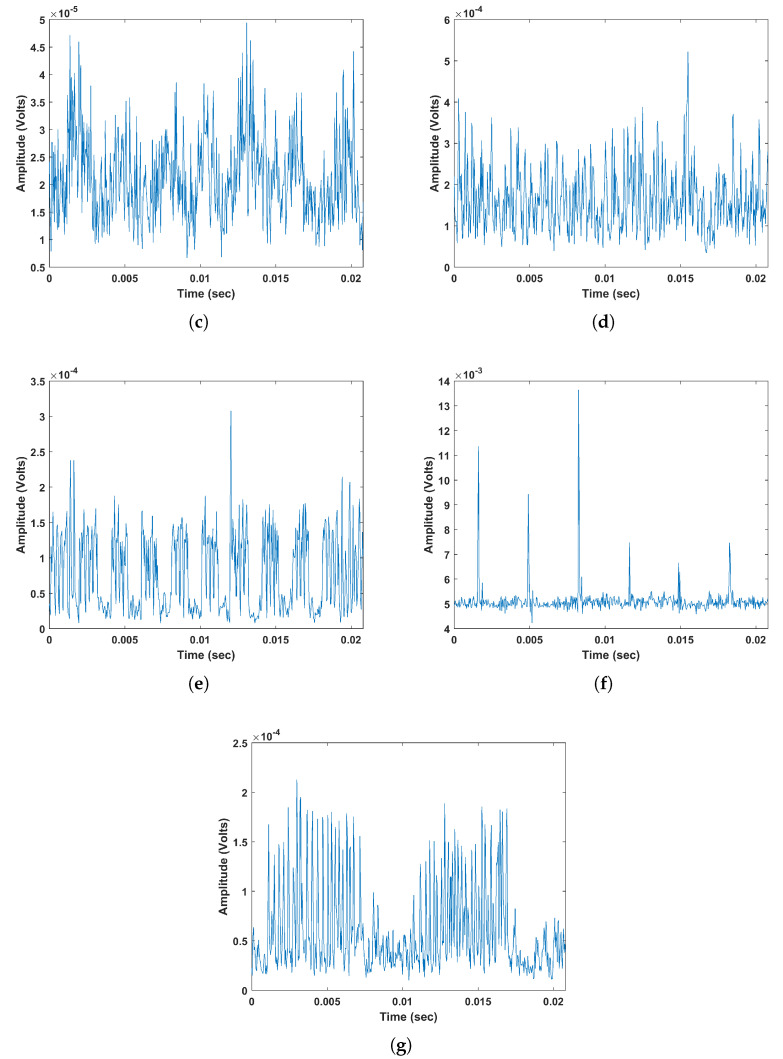
Time series signals of (**a**) Partial Discharge (PD) (**b**) Arcing (A) (**c**)Process Noise (PN) (**d**) Random Noise (RN) (**e**) Data Modulation (DM) (**f**) Exciter (E) (**g**) microSparking (mS).

EMI measurement technique, described in Section 2, has been used for data acquisition and expert analysis. EMI data was collected from ten different operating HV sites at various assets including motors, generators, cables, transformers and isolated phase-bus. A total of 7 EMI discharge types were identified within the data known and abbreviated as PD, Arcing (A), Process Noise (PN), Random Noise (RN), Data Modulation (DM), Exciter (E), and micro Sparking (mS). Table 1 shows the discharge sources identified within each site, and Figure 4 shows example time series from each signal type. It is important to highlight that the primary contribution in this paper is about classification of the different discharges and not trending of their severity levels. Yet, when the fault is classified by the algorithm, trending could be performed, for instance, increase in repetition rate and magnitude level, can be assessed [17,39].

### 4.2. Application of Feature Extraction and Classification

The collected and labelled EMI signals were split into segments of 4000 samples for ease of feature computation. PE, WPE, DE and SE measures were applied to each segment which significantly extracts important characteristics of each EMI source while potentially reducing data dimension for the classification algorithms. A total of *N* × 4 instances were implemented in MCSVM and RF classifiers. Training of each classifier is performed by introducing the training data set, with its associated labels, to the classifier. This results in a trained model which can be tested by presenting unseen data set without labels. The label for each testing instance are predicted by the model, and then compared to the true labels provided by “EMI experts”. The classifiers performance is obtained by calculating the accuracy (acc) %, precision (pr), recall (*r*) and F-measure (*F*) defined in Equations (48)–(51) respectively, where tp=true positives, fp= false positives, fn= false negatives and tpr= total predictions. Precision measures the level of exactness, whereas recall indicates the level of completeness within the classifier. F-measure is the harmonic mean of precision and recall which denotes the balance between them. A high value of these measures is preferable and the maximum performance has a value of 1. The precision of each class is presented in the confusion matrix (bottom row of matrices) and recall is shown in the last column accordingly.

(48) $$acc=\frac{tp}{tpr}\times 100$$

(49) $$pr=\frac{tp}{tp+fp}$$

(50) $$r=\frac{tp}{tp+fn}$$

(51) $$F=2\times \frac{pr\times r}{pr+r}$$

In this paper ten fold cross-validation approach is utilised to evaluate performance consistency. This involves training the classifier ten times, each with a different 90% of the data instances. The remaining instances are used for testing the classifiers.

## 5. Results

The average classification accuracy from each fold within individual sites is calculated and presented in Table 2. The proposed approach demonstrated high performance using both MCSVM and RF classifiers, while both strongly compete with each other. It is observed that in 8 of the sites (sites 1 to 3 and 5 to 9) both classifiers perform similarly well with high accuracy, but one marginally outperforms the other, with the exception of site 7 using RF where the lowest accuracy is observed. Notably, in sites 4 and 10 both classifiers achieve the top classification accuracy of 100%.

To further investigate and understand the lower performance results i.e., accuracy <98%, considering that a performance above 98% is excellent, the confusion matrix is calculated. It is important to visualise the classification algorithm’s performance from the confusion matrix aspect, especially for multi-class problems, as the total classification accuracy on its own may be misleading. The confusion matrix provides an in depth understanding on the predictions and errors. It shows the number of correct and wrong predictions within each class and what classes are being confused. Further discussion on these results is detailed as follows.

**Figure 5:**
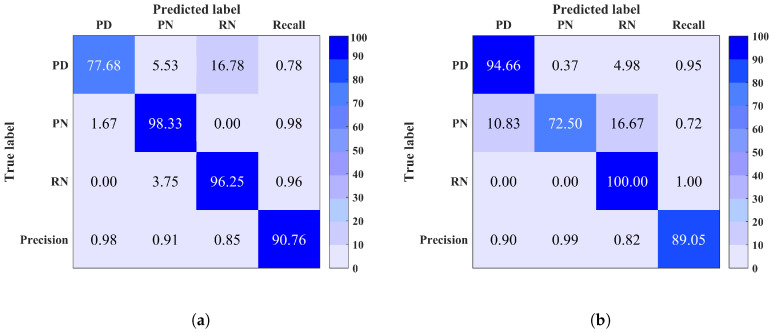
Confusion matrix of site 1 using (**a**) Multi-Class Support Vector Machine (MCSVM). (**b**) Random Forest (RF). Overall classification accuracy is shown in bottom right corner.

Site 1: Based on Figure 5, it is observed that although the total classification accuracy (shown in bottom right corner) is high, there is a confusion between PD, RN and PN. This could be due to the presence of high noise level within PD signals. In fact, the peaks of PD signals retrieved from high frequency in the EMI frequency spectrum tend to get attenuated and overwhelmed by noise. By referring to Figure 4 shows two example signals of PD and RN, in (a) and (b), collected from site 1. It is clear that PD signal is noisy which makes its classification more challenging, thus it could be easily confused with noise signals.

**Figure 6:**
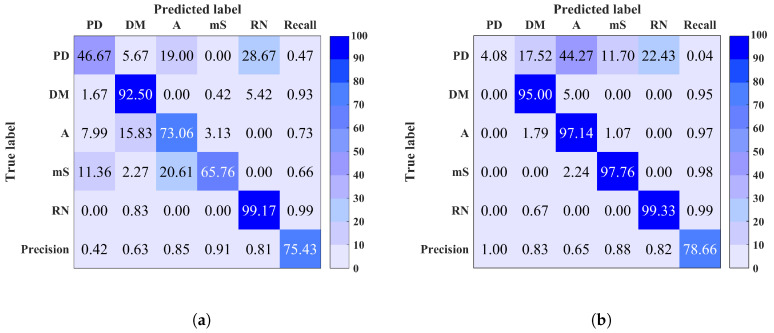
Confusion matrix of site 2 using (**a**) Multi-Class Support Vector Machine (MCSVM) (**b**) Random Forest (RF). Overall classification accuracy is shown in bottom right corner.

Site 2: The classification accuracy of this site is acceptable; however, it appears from Figure 6 that PD is mainly predicted as RN and A. It is likely that this confusion is due to the similar cause which was discussed for site 1.

Site 3: It is observed that this site contains two EMI classes only which are PD and E. Therefore the classification accuracy is affected by the confusion of these two classes only. This could be due to the similarity between PD and E signal nature i.e., narrow pulses shape.

**Figure 7:**
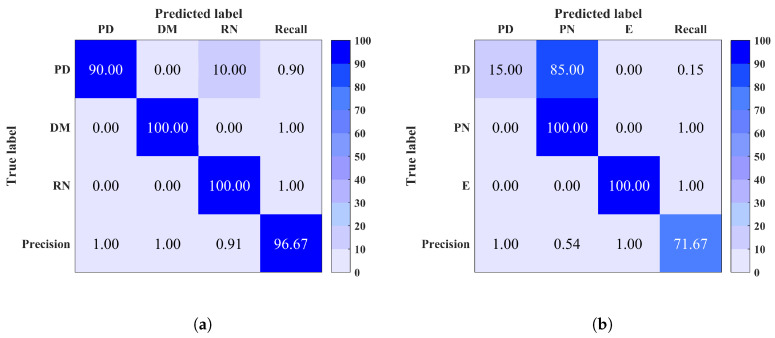
Confusion matrix of (**a**) site 5 (**b**) site 7 using Random Forest (RF). Overall classification accuracy is shown in bottom right corner.

Site 5: The obtained total classification accuracy for this site is very high using both classifiers. The confusion matrix in Figure 7a shows that the loss in classification accuracy is due to the prediction of some PD instances as RN. This is similar to the case of site 1 and 2.

Site 7: The confusion matrix of this site, illustrated in Figure 7b, explains the low classification accuracy such that PD instances were predicted as PN. Again, this is a similar case to sites 1, 2 and 5, where noisy PD signals are confused with noise. Note that for each site it is observed that the precision and recall are directly proportional to the classification rate within each class.

As shown above, noise could be a potential issue that may disrupt the classification performance. To overcome this issue, denoising using ALIF-TV technique is implemented as preprocessing.

**Figure 8:**
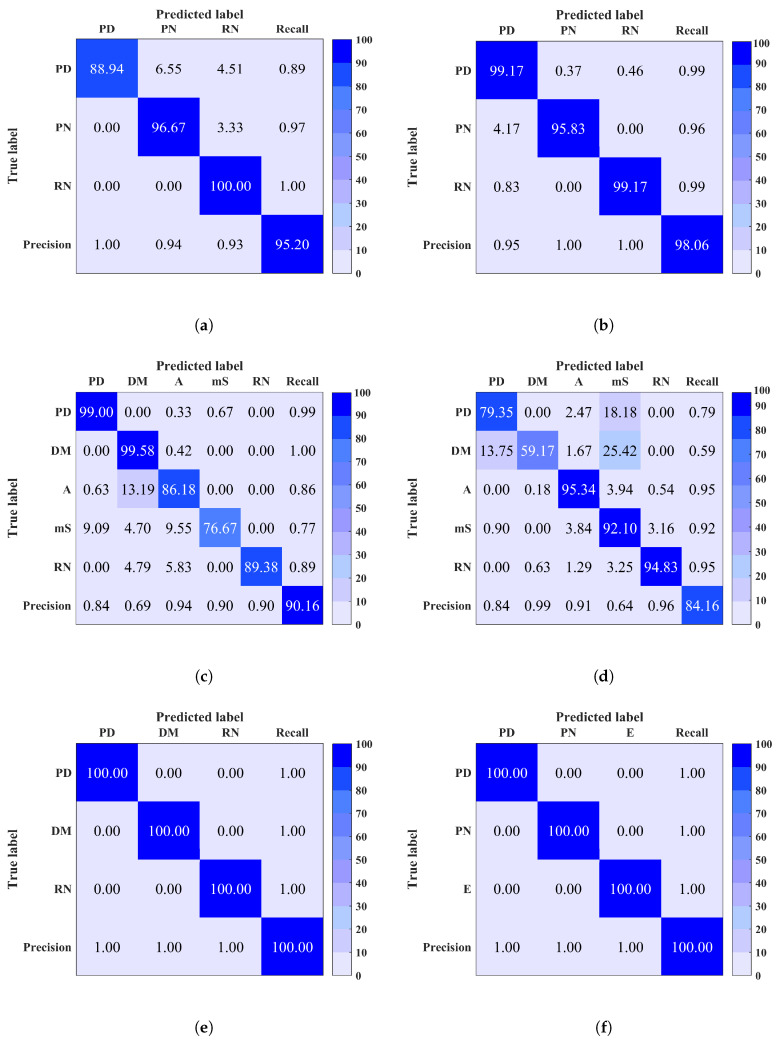
Confusion matrix of site 1 (**a**) using Multi-Class Support Vector Machine (MCSVM) (**b**) Random Forest (RF), site 2 (**c**) using MCSVM (**d**) using RF, and (**e**) site 5 (**f**) site 7 using RF, after denoising. Overall classification accuracy is shown in bottom right corner.

Classification results after denoising of any input signal with PAPR < 15 dB are shown in Figure 8. A remarkable improvement in classification performance is achieved after applying denoising in each site (1, 2, 5 and 7). A total accuracy improvement of 4–9% was achieved in site 1 for both MCSVM and RF respectively. In site 2, an improvement of 5–15% for RF and MCSVM respectively, was obtained. The maximum accuracy of 100% was obtained in sites 5 and 7. Furthermore, an improvement in precision, recall and F-measure was also observed in each site. Table 3 summarises the average accuracy, precision, recall and F-measure for the sites to which denoising was applied, using both classifiers. The difference in performance between denoised and noisy signals classification is clear. In summary, denoising should be considered for a better prediction as it minimises the confusion between noise signals (RN and PN) and the noisy discharge sources signals.

A second case of classification was studied where the data from all ten sites was mixed. Classification results employing both MCSVM and RF classifiers, for the raw as well as denoised signals, are presented in Table 4. It is observed that MCSVM performs better in this case, particularly after denoising.

## 6. Conclusions

This paper introduces the application of four entropy measures, called PE, WPE, DE and SE, as robust feature extraction techniques in the classification of 7 different EMI signal types by means of two popular classification algorithms known as RF and MCSVM. The developed model provides an intelligent system that captures an expert’s knowledge on electrical machine condition assessment using the EMI measurement technique. The performance of both classifiers demonstrated an overall good classification accuracy. However, it was found that noise affects the prediction results. As a solution to this issue, a denoising approach was proposed for the noisy signals which are then selected based on their PAPR. Denoising is implemented prior to feature extraction and classification. This approach achieved an improvement in classification results. This work has successfully demonstrated the applicability of Entropy-based methods within EMI HV condition monitoring. The application of other Entropy-based methods, such as Tsallis and Renyi will be considered in future work. In summary, the proposed feature extraction method transfers the measured complex time series signals to a simple low dimension data set which facilitates the classification process. This work could be considered in future developments of an automatic condition monitoring instrument for industrial applications, based on the fact that entropy measures are easy and simple to compute.

## Figures and Tables

**Table 1:** Identified discharge sources per site.

Site	Discharge Source
**1**	PD, RN, PN
**2**	mS, DM, RN, PD, A
**3**	PD, E
**4**	PD, E
**5**	RN, DM, PD
**6**	RN, DM, E, PD, mS
**7**	PN, E, PD
**8**	PD, E
**9**	PN, E, PD
**10**	PD, E

**Table 2:** Classification accuracy (rounded) results using Multi-Class Support Vector Machine (MCSVM) and Random Forest (RF).

Site		1	2	3	4	5	6	7	8	9	10
Accuracy %	MCSVM	91	75	91	100	96	99	100	99	100	100
	RF	89	79	92	100	97	98	72	100	99	100

**Table 3:** Average classification performance measures before and after denoising using MCSVM/RF.

Before Denoising					
	Site	1	2	5	7
	Accuracy %	91/89	75/79	96/97	100/72
	Precision	0.91/0.90	0.72/0.84	0.96/0.97	1/0.85
	Recall	0.91/0.89	0.76/0.79	0.97/0.96	1/0.72
	F-measure	0.91/0.90	0.74/0.81	0.96/0.96	1/0.78
**After denoising**					
	Accuracy %	95/98	90/84	100/100	100/100
	Precision	0.96/0.98	0.85/0.87	1/1	1/1
	Recall	0.98/0.98	0.90/0.84	1/1	1/1
	F-measure	0.97/0.98	0.87/0.85	1/1	1/1

**Table 4:** Average classification performance measures for all sites before and after denoising using MCSVM/RF.

Before Denoising	Accuracy %	Precision	Recall	F-Measure
	77/73	0.83/0.77	0.77/0.73	0.78/0.72
**After denoising**				
	91/66	0.91/0.79	0.91/0.66	0.91/0.65
